# Supplementary material for: An Unnatural Amino Acid-Regulated Growth Controller Based on Informational Disturbance
Source: Life (Basel). 2021 Sep 5;11(9):920. doi: 10.3390/life11090920 (PMC8467816; doi:10.3390/life11090920)
Supplement: Supplementary file 1 [file life-11-00920-s001.zip › life-1311832-supplementary.pdf]

Supporting Information

An unnatural amino acid-regulated growth controller based on informational disturbance

Yusuke Kato

Institute of Agrobiological Sciences, National Agriculture and Food Research Organization (NARO), Oowashi 1 - 2, Tsukuba, Ibaraki 305 - 8634, Japan; kato@affrc.go.jp; Tel.: +81 - 29 - 838 - 6059

Figure S1. Nucleotide sequence of the plasmid pTK2-1 ZLysRS1 with a modified anticodon. “XYZ”, highlighted in yellow-green, represents the anticodon sequence of the $tRNA^{pyl}_{complementary\ ZYX}$. pTK2-1 ZLysRS1 was originally constructed using pACYC184. The efficiency and specificity of the ZK-incorporation system have been reported [1,2]..

pTK2-1 ZLysRS1(tRNA$^{Pyl}_{XYZ}$)

1 ttggtagctc agagaacctt cgaaaaaccg ccctgcaagg cggttttttc gttttcagag

p15A ori.(continued)

61 caagagatta cgcgcagacc aaaacgatct caagaagatc atcttattaa tcagataaaa

121 tatttctaga tttcagtgca atttatctct tcaaatgtag cacctgaagt cagccccata

181 cgatataagt tgtaattctc atgtttgaca gcttatcat cgataagctt taatgcggta

241 gtttatcaca gttaaattgc taacgcagtc aggcaccgtg tatgaaatct aacaatgcgc

301 tcatcgtcat cctcggcacc gtcaccctgg atgctgtagg cataggcttg gttatgccgg

361 tactgccggg cctcttgcgg gatatcgtcc attccgacag catcgccagt cactatggcg

421 tgctgctagc gctatatgcg ttgatgcaat ttctatgcgc acccgttctc ggagcactgt

481 ccgaccgctt tggccgccgc ccagtcctgc tcgcttcgct acttggagcc actatcgact

541 acgcgatcat ggcgaccaca cccgtcctgt gcatcctcta cgccggacgc atcgtggccg

601 gcatcaccgg cgccacaggt gcggttgctg gcgcctatat cgccgacatc accgatgggg

661 aagatcgggc tcgccacttc gggctcatga gcgcttgttt cggcgtgggt atggtggcag

721 gccccgtggc cgggggactg ttgggcgcca tctccttgca tgcaccattc cttgcggcgg

781 cggtgctcaa cggcctcaac ctactactgg gctgcttcct aatgcaggag tcgcataagg

841 gagagcgtcg aagctttaat gcggtagttt atcacagtta aattgctaac gcagtcaggc

901 accgtgtatg aaatctaaca atgcgctcat cgtcatcctc ggcaccgtca ccctggatgc

961 tgtaggcata ggcttggtta tgccggtact gccgggcctc ttgcgggata tcgtccattc

1021 cgacagcatc gccagtcact atggcgtgct gctagcatcg acgagtctgg ctttgacatt

trpS regulatory region →

1081 cgactagaag tggacggtgg cgtgaaggtg aacaacattg gcgaaatcgc tgcggcgggc

1141 gcggatatgt tcgtcgccgg ttcggcaatc ttcgaccagc cagactacaa aaaagtcatt

1201 gatgaaatgc gcagtgaact ggcaaaggta agtcatgaat aagtttgaag atattcgcgg

1261 cgtcgctttt gatcttgatg gtacgctggt cgacagtgct cctggtcttg ctgctgcggt

1321 agatatggcg ctgtatgcgc tggagttgcc cgtcgcaggt gaagaacgcg ttattacctg

1381 gattggtaac ggcgcagatg ttctgatgga gcgcgcattg acctggcgc gtcaggaacg

1441 tgcgactcag cgtaaaacaa tgggtaaacc gcccgttgat gacgacattc cggcagaaga

1501 acaggtacgt attctgcgta aactgttcga tcgctactat ggcgaggttg ccgaagaggg

1561 gacgtttttg ttcccgcacg ttgccgatac gttgggcgcg ttgcaggcta aaggcctgcc

1621 gctaggcctg gtcaccaaca aaccgacgcc gttcgtcgcg ccgctgctcg aagccttaga

1681 tatcgccaaa tacttcagcg cggtgattgg tggtgatgat gtgcaaaaca aaaaaccgca

1741 tccggacccg ctgttactgg tggctgagcg gatgggaatt gccccacaac agatgctgtt

1801 tgtcggcgac tcacgcaatg atattcaggc ggcaaaagcg gcaggttgcc catcagttgg

1861 cttaacctac ggatataact acggcgaggc tatcgatctc agccagcctg atgtaattta

1921 tcagtctata aatgaccttc tgcccgcatt agggcttccg catagcgaaa atcaggaatc

1981 gacatATGGA TAAAAAACCA CTAAACACTC TGATATCTGC AACCGGGCTC TGGATGTCCA

ZKRS →

2041 GGACCGGAAC AATTCATAAA ATAAAACACC ACGAAGTCTC TCGAAGCAAA ATCTATATTG

2101 AAATGGCATG CGGAGACCAC CTTGTTGTAA ACAACTCCAG GAGCAGCAGG ACTGCAAGAG
2161 CGCTCAAGCA CCACAAATAC AGGAAGACCT GCAAACGCTG CAGGGTTTCG GATGAGGATC
2221 TCAATAAGTT CCTCACAAAG GCAAACGAAG ACCAGACAAG CGTAAAAGTC AAGGTCGTTT
2281 CTGCCCCTAC CAGAACGAAA AAGGCAATGC CAAAATCCGT TGCGAGAGCC CCGAAACCTC
2341 TTGAGAATAC AGAAGCGGCA CAGGCTCAAC CTTCTGGATC TAAATTTTCA CCTGCGATAC
2401 CGGTTTCCAC CCAAGAGTCA GTTTCTGTCC CGGCATCTGT TTCAACATCA ATATCAAGCA
2461 TTTCTACAGG AGCAACTGCA TCCGCACTGG TAAAAGGGAA TACGAACCCC ATTACATCCA
2521 TGTCTGCCCC TGTTCAGGCA AGTGCCCCCG CACTTACGAA GAGCCAGACT GACAGGCTTG
2581 AAGTCCTGTT AAACCCAAAA GATGAGATTT CCCTGAATTC CGGCAAGCCT TTCAGGGAGC
2641 TTGAGTCCGA ATTGCTCTCT CGCAGAAAAA AAGACCTGCA GCAGATCTAC GCGGAAGAAA
2701 GGGAGAATTA TCTGGGGAAA CTCGAGCGTG AAATTACCAG GTTCTTTGTG GACAGGGGTT
2761 TTCTGGAAAT AAAATCCCCG ATCCTGATCC CTCTTGAGTA TATCGAAAGG ATGGGCATTG
2821 ATAATGATAC CGAACTTTCA AAACAGATCT TCAGGGTTGA CAAGAACTTC TGCCTGAGAC
2881 CCATGCTTGC TCCAAACCTT GCCAACTACC TGCGCAAGCT TGACAGGGCC CTGCCTGATC
2941 CAATAAAAAT TTTTGAAATA GGCCCATGCT ACAGAAAAGA GTCCGACGGC AAAGAACACC
3001 TCGAAGAGTT TACCATGCTG AACTTCTGCC AGATGGGATC GGGATGCACA CGGGAAAATC
3061 TTGAAAGCAT AATTACAGAC TTCCTGAACC ACCTGGGAAT TGATTTCAAG ATCGTAGGCG
3121 ATTCCTGCAT GGTCTTTGGG GATACCCTTG ATGTAATGCA CGGAGACCTG GAACTTTCCT
3181 CTGCAGTAGT CGGACCCATA CCGCTTGACC GGGAATGGGG TATTGATAAA CCCTGGATAG
3241 GGGCAGGTTT CGGGCTCGAA CGCCTTCTCA AGGTTAAACA CGACTTTAAA AATATCAAGA
3301 GAGCTGCAAG GTCCGGGTCT TACTATAACG GGATTTCTAC CAACCTGTAA ggatctgcat
3361 cgcaggatgc tgctggctac cctgtggaac acctacatct gtattaacga agcgctggca
3421 ttgaccctga gtgatttttc tctggtcccg ccgcatccat accgccagtt gtttaccctc
3481 acaacgttcc agtaaccggg catgttcatc atcagtaacc cgtatcgtga gcatcctctc
3541 tcgtttcatc ggtatcatta cccccatgaa cagaaatccc ccttacacgg aggcatcagt
3601 gaccaaacag gaaaaaaccg cccttaacat ggcccgcttt atcagaagcc agacattaac
3661 gcttctggag aaactcaacg agctggacgc ggatgaacag gcagacatct gtgaatcgct
3721 tcacgaccac gcatcaaaaa aaatccttag ctttcgctaa ggatctgcag TGGCGGAAAC
3781 CCCGGGAATC TAACCCGGCT GAACGGATTX YZAGTCCATT CGATCTACAT GATCAGGTTT
3841 CCGGATCCGT TACAAGTATT ACACAAAGTT TTTTATGTTG AGAATATTTT TTTGATgggg

← tRNA$^{pyl}_{XYZ}$ ← *lpp* promoter

3901 cgccacttat ttttgatcgt tcgctcaaag aagcggcgcc gtcgaccgat gcccttgaga
3961 gccttcaacc cagtcagctc cttccggtgg gcgcggggca tgactatcgt cgccgcactt
4021 atgactgtct tctttatcat gcaactcgta ggacaggtgc cggcagcgct ctgggtcatt
4081 ttcggcgagg accgctttcg ctggagcgcg acgatgatcg gcctgtcgct tgcggtattc
4141 ggaatcttgc acgccctcgc tcaagccttc gtcactggtc ccgccaccaa acgtttcggc
4201 gagaagcagg ccattatcgc cggcatggcg gccgaggtct gcctcgtgaa gaaggtgttg
4261 ctgactcata ccaggcctga atcgccccat catccagcca gaaagtgagg gagccacggt
4321 tgatgagagc tttgttgtag gtggaccagt tggtgatttt gaacttttgc tttgccacgg

4381 aacggtctgc gttgtcggga agatgcgtga tctgatcctt caactcagtt acgccccgcc
4441 ctgccactca tcgcagtact gttgtaattc attaagcatt ctgccgacat ggaagccatc
4501 acaaacggca tgatgaacct gaatcgccag cggcatcagc accttgtcgc cttgcgtata
4561 atatttgccc atggtgaaaa cgggggcgaa gaagttgtcc atattggcca cgtttaaatc
4621 aaaactggtg aaactcaccc agggattggc tgagacgaaa aacatattct caataaaccc
4681 tttagggaaa taggccaggt tttcaccgta acacgccaca tcttgcgaat atatgtgtag
4741 aaactgccgg aaatcgtcgt ggtattcact ccagagcgat gaaaacgttt cagtttgctc
4801 atggaaaacg gtgtaacaag ggtgaacact atcccatatc accagctcac cgtctttcat
4861 tgccatacgg aattccggat gagcattcat caggcgggca agaatgtgaa taaaggccgg
4921 ataaaacttg tgcttatttt tctttacggt ctttaaaaag gccgtaatat ccagctgaac
4981 ggtctggtta taggtacatt gagcaactga ctgaaatgcc tcaaaatgtt ctttacgatg
5041 ccattgggat atatcaacgg tggtatatcc agtgattttt ttctccattt tagcttcctt

← Cm^R

5101 agctcctgaa aatctcgata actcaaaaaa tacgcccggt agtgatctta tttcattatg
5161 gtgaaagttg gaacctctta cgtgccgatc aacgtctcat tttcgccaaa agttggccca
5221 gggcttcccg gtatcaacag ggacaccagg atttatttat tctgcgaagt gatcttccgt
5281 cacaggtatt tattcggcgc aaagtgcgtc gggtgatgct gccaacttac tgatttagtg
5341 tatgatggtg tttttgaggt gctccagtgg cttctgtttc tatcagctgt ccctcctgtt
5401 cagctactga cggggtggtg cgtaacggca aaagcaccgc cggacatcag cgctagcgga

p15A ori.

5461 gtgtatactg gcttactatg ttggcactga tgagggtgtc agtgaagtgc ttcatgtggc
5521 aggagaaaaa aggctgcacc ggtgcgtcag cagaatatgt gatacaggat atattccgct
5581 tcctcgctca ctgactcgct acgctcggtc gttcgactgc ggcgagcgga aatggcttac
5641 gaacggggcg gagatttcct ggaagatgcc aggaagatac ttaacaggga agtgagaggg
5701 ccgcggcaaa gccgtttttc cataggctcc gcccccctga caagcatcac gaaatctgac
5761 gctcaaatca gtggtggcga aacccgacag gactataaag ataccaggcg tttccccctg
5821 gcggctccct cgtgcgctct cctgttcctg cctttcggtt taccggtgtc attccgctgt
5881 tatggccgcg tttgtctcat tccacgcctg acactcagtt ccgggtaggc agttcgctcc
5941 aagctggact gtatgcacga acccccegtt cagtccgacc gctgcgcctt atccggtaac
6001 tatcgtcttg agtccaaccc ggaaagacat gcaaaagcac cactggcagc agccactggt
6061 aattgattta gaggagttag tcttgaagtc atgcgccggt taaggctaaa ctgaaaggac
6121 aagttttggt gactgcgctc ctccaagcca gttacctcgg ttcaaagag

Figure S2. Preliminary tests to elucidate the mode of action for anticodon sequences that exhibit strong growth inhibition. Anticodon sequences are shown as corresponding codon sequences. The anticodon CUA corresponding to UAG(stop-amber), which does not exhibit toxicity, was also tested as a control. In each photo, viable bacteria were detected as colonies after incubation in medium containing 3 mM ZK for 0 h (left) and 6 h (right). For detailed experimental procedures, see Materials and Methods. Uneven colony distribution is an artificial phenomenon caused by uneven inoculation.

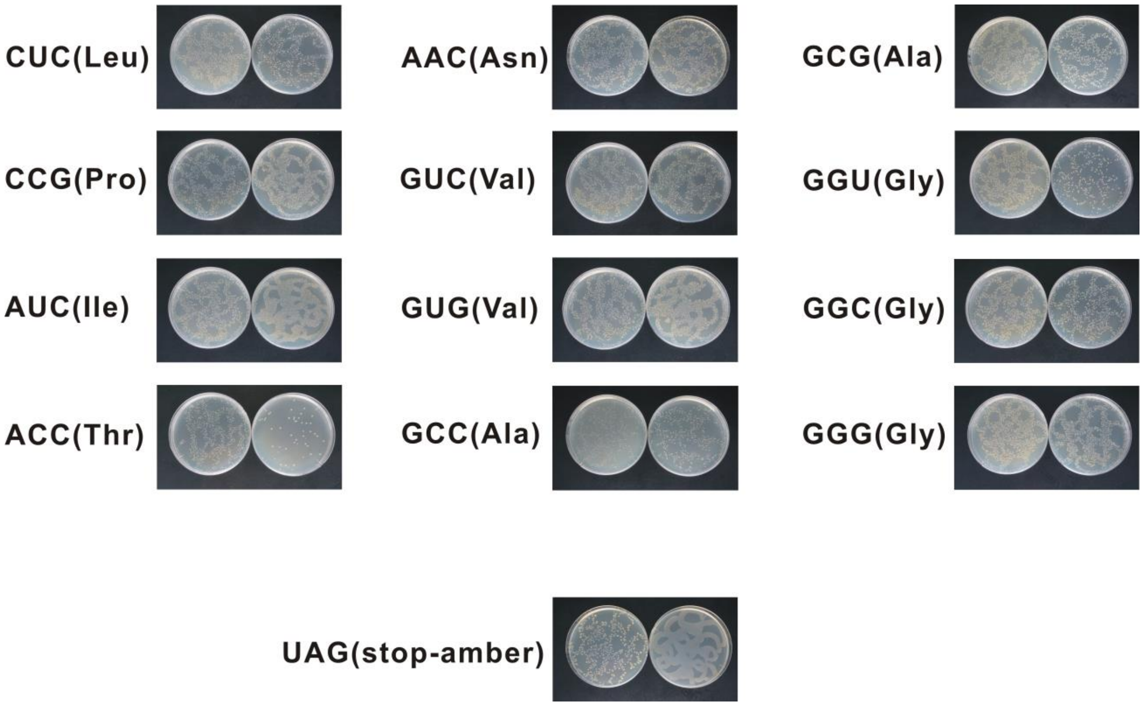
CUC(Leu)
AAC(Asn)
GCG(Ala)
CCG(Pro)
GUC(Val)
GGU(Gly)
AUC(Ile)
GUG(Val)
GGC(Gly)
ACC(Thr)
GCC(Ala)
GGG(Gly)
UAG(stop-amber)

Talbe S1. Detailed data on anticodon sequence-dependency of growth inhibition. Anticodon sequences are shown as corresponding codon sequences. Amino acids assigned to the codons are also shown. After bacterial culture reached OD_{590} = 0.2-0.3 (approximately 3 h), the OD_{590} values of tested cultures were measured. The degree of growth inhibition was evaluated using the relative value of OD_{590} of the culture in the presence of 3 mM ZK to OD_{590} of the culture in the absence of ZK. Data are shown as the mean and s.d. of three biological replicates. The relative toxicity of GN_1N_2 and CN_1N_2 anticodons was evaluated as “higher”, “almost equal” and “lower”. Statistical analyses were performed using one-way ANOVA in Excel ver.14.0, with $p < 0.05$ considered significant.

		Relative growth (%)	S.D.
TTT	Phe	44.3	4.7
TTC	Phe	17	1.7
TTA	Leu	37.3	4
TTG	Leu	10	2.6
TCT	Ser	8.7	0.6
TCC	Ser	9.3	2.1
TCA	Ser	46.7	3.5
TCG	Ser	11	1.7
TAT	Tyr	82.3	4.9
TAC	Tyr	15.7	2.1
TAA	stop−ochre	46.7	2.3
TAG	stop−amber	100	3
TGT	Cys	88.7	3.1
TGC	Cys	18.7	3.2
TGA	stop−opal	101.3	2.3
TGG	Trp	15	0
CTT	Leu	13	0
CTC	Leu	−6.3	0.6
CTA	Leu	40.7	2.1
CTG	Leu	14.3	0.6
CCT	Pro	41.7	2.9
CCC	Pro	10.3	3.8
CCA	Pro	18	2.6
CCG	Pro	1.3	2.3
CAT	His	38.7	2.1
CAC	His	18	1.7
CAA	Gln	43.3	0.6
CAG	Gln	19.7	0.6
CGT	Arg	4.3	2.3
CGC	Arg	12	1.7
CGA	Arg	66.3	2.1
CGG	Arg	67.7	1.2

		Relative growth (%)	S.D.
ATT	Ile	43.7	1.5
ATC	Ile	2.3	2.1
ATA	Ile	55.7	4.9
ATG	Met	15.3	2.1
ACT	Thr	23.3	2.1
ACC	Thr	−4	3.5
ACA	Thr	44.3	2.1
ACG	Thr	6.3	3.2
AAT	Asn	96.7	2.9
AAC	Asn	4	0
AAA	Lys	78	5.6
AAG	Lys	60	3
AGT	Ser	55	3.6
AGC	Ser	38.7	4.2
AGA	Arg	101.7	2.9
AGG	Arg	82	4.6
GTT	Val	9	0
GTC	Val	−2.7	2.3
GTA	Val	19	0
GTG	Val	−1	1.7
GCT	Ala	25.3	2.1
GCC	Ala	−8.7	2.1
GCA	Ala	14	1
GCG	Ala	−1	1.7
GAT	Asp	22.7	1.5
GAC	Asp	9.3	2.3
GAA	Glu	27.3	2.3
GAG	Glu	44.7	2.1
GGT	Gly	−1.7	2.9
GGC	Gly	−3.3	2.9
GGA	Gly	26.3	2.1
GGG	Gly	2.7	2.3

Relative growth inhibitory effect of (G/C)N_1N_2 anticodon

Higher
Almost equal
Lower
Not included in this analysis

References

1. Yanagisawa, T.; Ishii, R.; Fukunaga, R.; Kobayashi, T.; Sakamoto, K.; Yokoyama, S. Multistep engineering of pyrrolysyl-tRNA synthetase to genetically encode N^{ε}-(o-Azidobenzyloxycarbonyl)lysine for site-specific protein modification. *Chem. Biol.* **2008**, *15*, 1187-1197.
2. Kato Y. Tight Translational Control Using Site-Specific Unnatural Amino Acid Incorporation with Positive Feedback Gene Circuits. *ACS Synth. Biol.* **2018**, *7, 8,* 1956–1963.
